# Maturogenesis of a Complicated Crown Fracture: A Case Report with 8 Years Follow-Up

**Published:** 2007-04-01

**Authors:** Masoud Parirokh, Shahla Kakoei, Ali Eskandarizadeh

**Affiliations:** 1 *Department of Endodontics, Dental School, Kerman University of Medical Sciences, Kerman, Iran*; 2 *Department of Oral Medicine, Dental School, Kerman University of Medical Sciences, Kerman, Iran*; 3 *Department of Restorative Dentistry, Dental School, Kerman University of Medical Sciences, Kerman, Iran*

**Keywords:** Apexosgenesis, Crown Fracture, Maturogenesis, Partial Pulpotomy

## Abstract

This report describes a case of a 7 years old girl who suffered from complicated crown fracture of right mandibular central incisor because of a bicycle accident. For the tooth partial pulpotomy with calcium hydroxide, capping was performed in order to achieve apexogenesis and the tooth was restored with a double-seal of glass ionomer cement and composite resin. The patient was reviewed over 8 years. The tooth showed continued root development and complete apex formation.

## INTRODUCTION

Facial injuries occur more frequently in children than adults and are usually as a result of sports activities, falls, car accidents, fights and intentional assaults (1). Blows to the face often affect the teeth and especially the maxillary incisors because of their normal labial projection in relation to the mandibular incisors, most of the time leading to crown damages (2). However, sometimes mandibular teeth may suffer from traumatic injuries (3). Complicated crown fractures result in the exposure of the pulps. Crown fractures with pulp exposure represent 0.9% to 13% of all traumatic injuries of the teeth (4). The majority of these injuries occur in recently erupted or in young permanent teeth with immature roots (5). Accordingly, treatment is oriented towards preserving pulp function (1). Apexogenesis is a vital pulp therapy procedure performed to encourage continued physiological development and formation of root ends; frequently used to describe vital pulp therapy performed to encourage the continuation of this process (6).

Maturogenesis is the recent attractive term and has been defined as the physiological root development, not restricted to the apical segment (7). The continued deposition of dentin occurs throughout the length of the root, providing greater strength and resistance to fracture.

The key factor in determining prognosis after any pulp exposure is minimizing the bacterial invasion to the pulp (8). Therefore, provision of a hermetic seal over exposed pulps to minimize invasion by bacteria and the removal of infected pulpal tissue as soon as possible following injuries is critical (8). In this article a case of complicated crown fracture is presented which was managed properly and followed-up for 8 years.

## CASE REPORT

**Figure 1:**
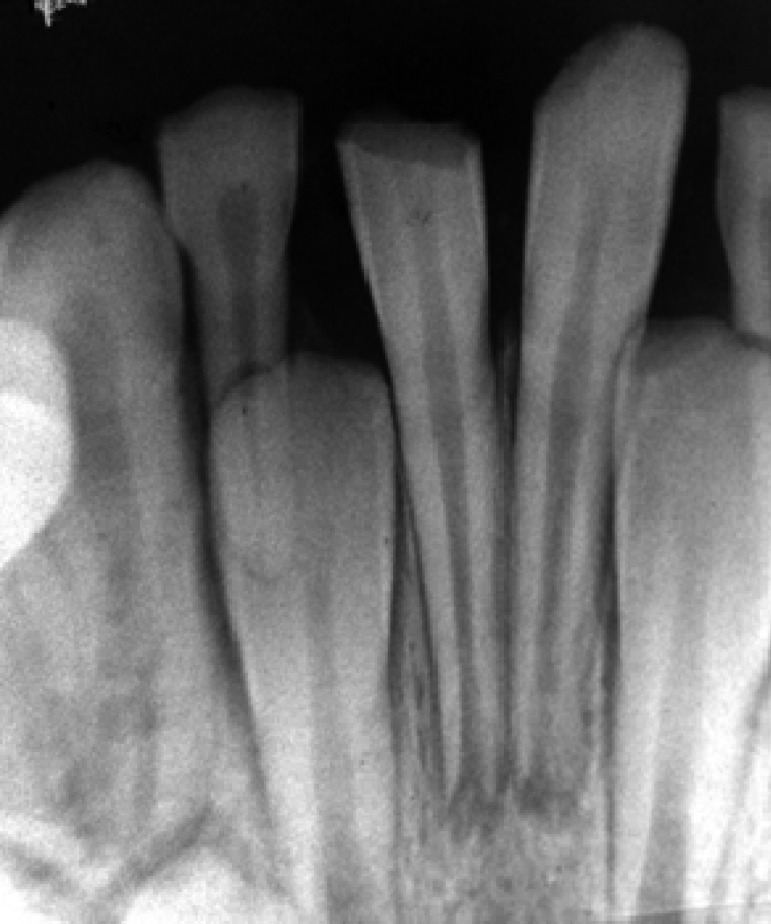
Radiographic image of right and left mandibular incisors after trauma

A 7-year-old girl attended because of trauma that caused complicated crown fracture in the right central mandibular incisor. The patient referred 4 hours after accident. Her medical history was non-contributory. No spontaneous pain was reported by the patient and her main complaint was sensitivity to palpation of the fractured tooth.Clinical examination showed a complicated crown fracture with pulpal exposure on the right central mandibular incisor and an uncomplicated crown fracture on the left central incisor. Radiographic image (Figure 1) demonstrated the immature apex of fractured tooth.

**Figure 2:**
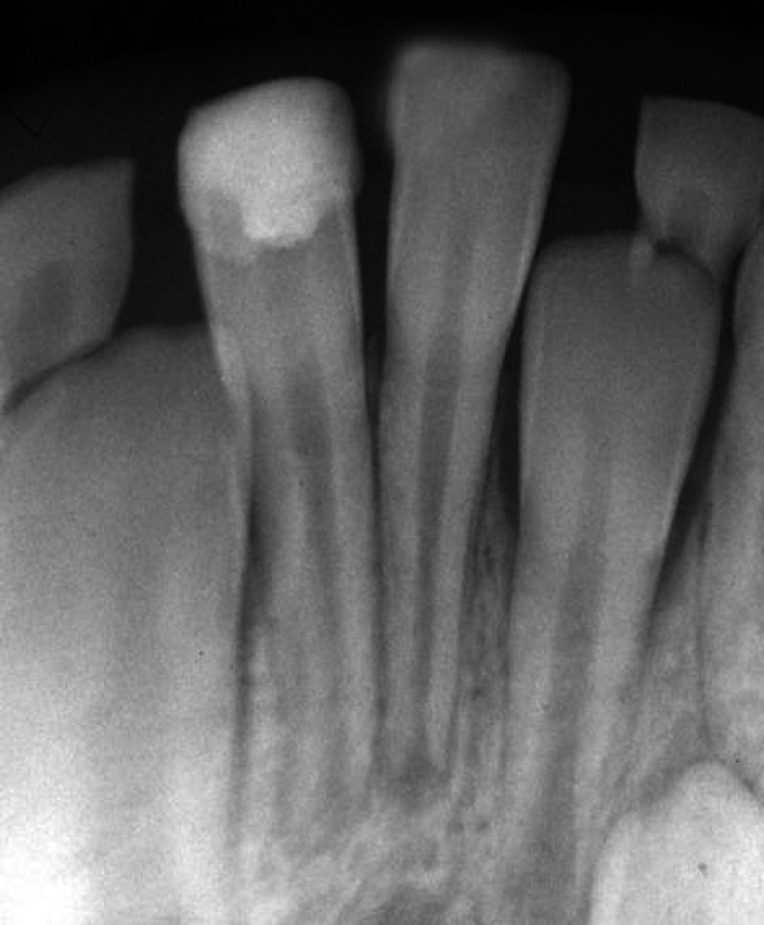
X-ray image of the right central incisor after performing partial pulpotomy

However, there was not any sign of root fracture. Under local anesthesia using 2% lidocanie, 1:80000 epinephrine (Darupakhsh, Tehran, Iran), and rubber dam, partial pulpotomy was performed in the right central incisor using a diamond bur (Diatech, Heerbrugg, Switzerland). The area was rinsed with 2.5% sodium hypochlorite (Golrang-Tehran, Iran). After that, the pulp was covered with pure calcium hydroxide powder (Merck, Darmstadt, Germany) that was mixed with physiologic saline (Samen Industries, Mashhad, Iran) to a very dry thick mixture and which was condensed with a light vertical pressure to a thickness of 1–2 mm. The right central incisor tooth was restored with glass ionomer cement (Fuji, Japan). The left central incisor was restored with light cure composite resin (King Dental Corp., USA). After 1 week, part of the glass-ionomer was removed and the tooth was restored with light-cured composite resin (Figure 2). Six months later, the patient was followed up for clinical and radiographic evaluation.

**Figure 3:**
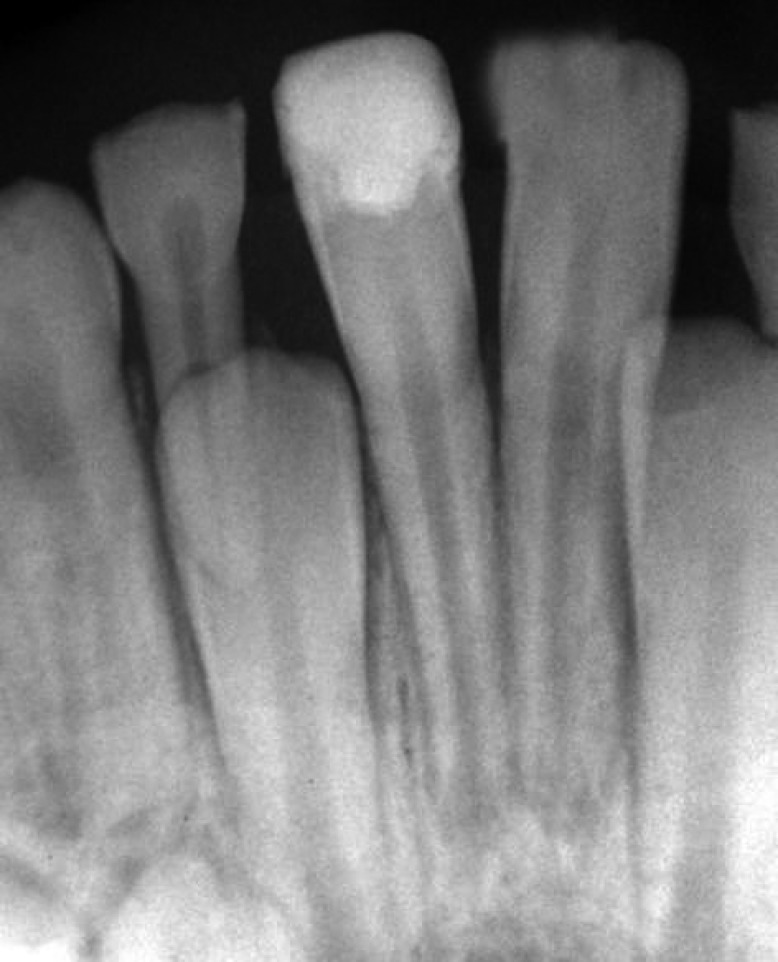
Radiographic image of the central mandibular incisors 6 months after treatment

**Figure 4:**
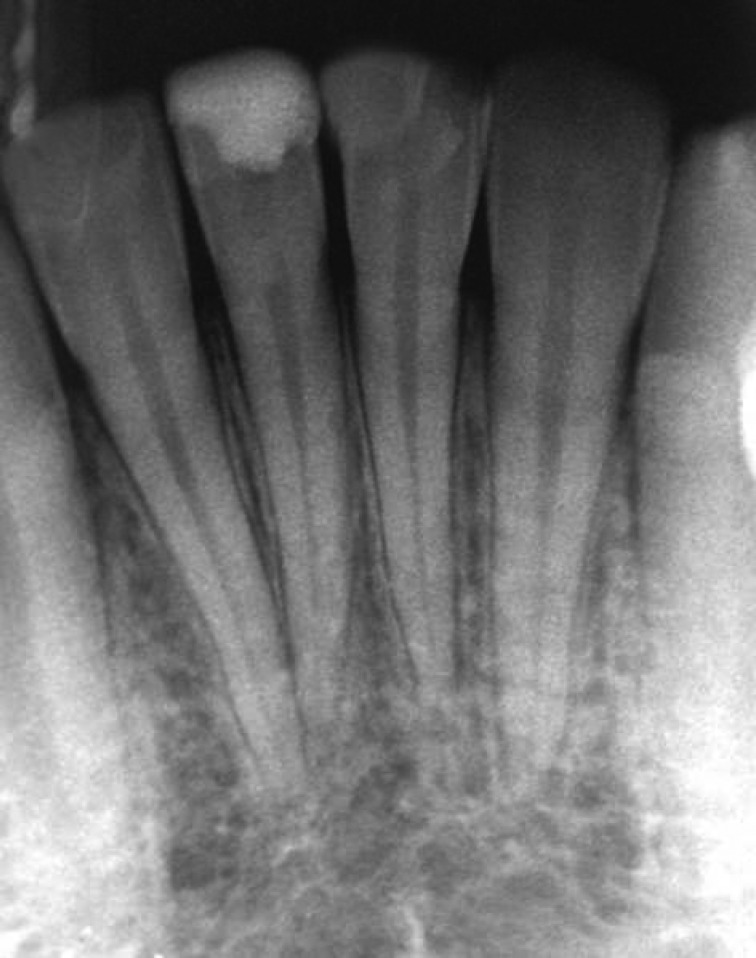
Radiographic image of the teeth 8 years after treatment

Radiographic images showed root development (Figure 3). Unfortunately, the patient did not register for follow ups. After 8 years, however, the patient came back to the office to take advice because of the discoloration of composite resin restoration. The patient was symptom-free and comfortable. A radiographic image (Figure 4) showed that the tooth had favorably developed and there was no evidence of either resorption (internal or external) or periradicular pathosis. Patient was referred to a restorative dentist for esthetic consultation.

## DISCUSSION

Complicated crown fractures usually result exposures that permit salivary rinsing and prevent impaction of contaminated debris (9-10). However, bacterial contamination from plaque, contaminated debris over the exposed pulp or leakage of a deficient temporary restoration allows bacteria to settle in the pulp. Therefore, the provision of a well intentioned but ultimately poor quality temporary restoration over an exposed pulp may actively weakened the tooth’s prognosis, emphasizing the importance of optimum treatment quality from the outset. The key factor in determining prognosis after any pulp exposure is minimizing the bacterial invasion of the pulp (2). Therefore, provision of a hermetic seal over exposed pulps is critical in order to minimize bacterial invasion and remove the infected pulpal tissue as soon as possible.

Today’s composite resins are the material of choice for crown restoration after complicated and uncomplicated crown fracture teeth as it is used for this case (11).

Cvek (12) found pulpotomies which remove the superficial area of infected pulp to have very high success rates, regardless of the size of the exposure or the time elapsed since injury, providing the pulp was previously healthy. Teeth with substantial treatment delay healed as well as teeth treated immediately, indicating that duration of post-injury time, by itself, is not a contraindication for undertaking a pulpotomy (12). Maintenance of vitality will exploit the full potential of the pulp for dentin deposition and will produce a stronger mature root that is better able to withstand fracture (2). Factors which influence treatment planning when encountering teeth with pulpal exposure include the degree of infection and inflammation in the pulp space, rather than the size or duration of pulp exposure (13). For traumatic exposures in young asymptomatic immature teeth, a direct pulp cap or partial pulpotomy are the treatments of choice (14). For above mentioned reasons partial pulpotomy was preferred.

In this case 2.5% sodium hypochlorite was used for rinsing the capping area after coronal pulp removing. The histological extent and degree of inflammation cannot be accurately predicted clinically. Studies have demonstrated that optimum hemorrhage control is essential for successful outcome of direct pulp capping regardless of the material used (14,15). Sodium hypochlorite in concentrations of 2.5–5.25%, in addition to being ideal for hemorrhage control when placed on an exposed pulp, also provides asepsis, chemical amputation of the blood clot and fibrin and removal of damaged cells and operative debris from the mechanical exposure and subjacent pulp. Optimum hemostasis will also help achieving the goal of bacteriometic seal. Other studies showed that sodium hypochlorite does not impair or retard the cellular healing of exposed pulps and is not inhibitory to the biological mechanisms of Odontoblast-like cell or dentin bridge formation (16). In addition, it can be used for removal of residual microbial flora, which can be a major deterrent in healing of exposed pulp. Above mentioned reasons are good rationales for rinsing with sodium hypochlorite in this case.

Different materials have been introduced as pulp capping agents such as calcium hydroxide (17), mineral trioxide aggregate (MTA) (18-20), hydroxyapatite (21) etc. Of these, calcium hydroxide is the most popular one (21). However, many research studies have shown deficiencies of calcium hydroxide include poor adherence to dentin, inability to form a long-term seal against bacterial microleakage and a porous dentinal bridge formation (22). Animal studies producing promising results when MTA compared with calcium hydroxide (19,20). Pulpal tissue preservation, uniform calcified bridge formation, rate of bridge formation and providing excellent seal make MTA a new choice for pulpal maturogenesis (22). In this case we used calcium hydroxide because at the time of treatment MTA had not been marketed yet.

## CONCLUSION

The result of this case report showed that if the treatment performed soon with a reasonable restoration over pulp capping agent, the success would be met by the clinician.
